# Delusional parasitosis: the importance of a multidisciplinary approach

**DOI:** 10.1192/j.eurpsy.2024.1561

**Published:** 2024-08-27

**Authors:** M. Calvo Valcárcel, G. Guerra Valera, M. A. Andreo Vidal, O. Martín Santiago, M. Lorenzo Hernando, M. P. Pando Fernández, P. Martinez Gimeno, M. D. L. Á. Guillén Soto, B. Rodríguez Rodríguez, N. Navarro Barriga, M. Fernández Lozano, M. J. Mateos Sexmero, C. De Andrés Lobo, M. D. C. Vallecillo Adame, T. Jimenez Aparicio, A. Monllor Lazarraga, M. Ríos Vaquero, L. Rojas Vázquez, L. Sobrino Conde, A. Aparicio Parra, G. Lorenzo Chapatte

**Affiliations:** Psychiatry, Hospital Clínico Universitario de Vallaldolid., Valladolid, Spain

## Abstract

**Introduction:**

Delusional parasitosis, also known as delusional infestation or Ekbom’s syndrome, is a rare psychotic disorder characterized by the false belief that a parasitic skin infestation exists, despite the absence of any medical evidence to support this claim. These patients often see many physicians, so a multidisciplinary approach among clinicians is important. Many patients refuse any treatment due to their firm belief that they suffer from an infestation, not a psychiatric condition, so it is crucial to gain the trust of these patients.

**Objectives:**

The comprehensive review of this clinical case aims to investigate Ekbom syndrome, from a historical, clinical and therapeutic perspective.

**Methods:**

Literature review based on delusional parasitosis.

**Results:**

A 65-year-old woman comes to the psychiatry consultation referred by her primary care physician concerned about being infested by insects that she perceives through scales on her skin for the last three months. She recognizes important impact on her functionality. She is also convinced that her family is being infected too. As psychiatric history she recognizes alcohol abuse in the past (no current consumption) and an episode of persecutory characteristics with a neighbor, more than ten years ago. On psychopathological examination, she shows delusional ideation of parasitosis, with high behavioral repercussions, cenesthetic and cotariform hallucinations, as well as feelings of helplessness and anger. Treatment with Pimozide was started and the patient was referred to dermatology for evaluation, a plan she accepted. Her primary care physician and dermatology specialist were informed about the case and the treatment plan. In the recent reviews, the patient is calmer, however, despite the corroboration of dermatology and in the absence of organic lesions in cranial CT, she is still unsatisfied with the results, remaining firm in her conviction of infestation. It was decided to start treatment with atypical neuroleptics (Aripiprazole), with progressive recovery of her previous functionality.

**Conclusions:**

Despite the increase in the number of studies in recent years, there are still few studies on this type of delirium. The female:male ratio varies in the bibiliography (between 2:1 and 3:1). The onset is usually insidious, generally appearing as a patient who comes to his primary care physician convinced of having parasites in different skin locations. It is usual to observe scratching lesions or even wounds in search of the parasite. In the past, the most used and studied treatment was Pimozide. Currently the treatment of choice is atypical neuroleptics due to their lower side effects. The latest reviews on the prognosis of this disorder show data with percentages of complete recovery between 51% and 70%, and partial responses between 16.5% and 20%. Finally, for a good diagnosis and therapeutic management, it is important to achieve a multidisciplinary approach.

**Disclosure of Interest:**

None Declared

